# Health Effects of Peptides Extracted from Deer Antler

**DOI:** 10.3390/nu14194183

**Published:** 2022-10-08

**Authors:** Peijun Xia, Dongyue Liu, Yingying Jiao, Zhaoguo Wang, Xi Chen, Shuo Zheng, Jiayuan Fang, Linlin Hao

**Affiliations:** 1 College of Animal Science, Jilin University, Changchun 130062, China; 2 Jilin Province Guoda Biological Engineering Co., Ltd., Changchun 130102, China

**Keywords:** deer antler, peptides, natural medicines, health-promoting properties, anti-inflammatory, antioxidation, bone protecting, anti-neurodegeneration

## Abstract

Deer antler is widely used as a nutraceutical in Asian countries. In the past decades, deer antler peptides (DAPs) have received considerable attention because of their various biological properties such as antioxidant, anti-inflammatory, anti-bone damage, anti-neurological disease, anti-tumor and immunomodulatory properties. This review describes the production methods of DAPs and the recent progress of research on DAPs, focusing on the physiological functions and their regulatory mechanisms.

## 1. Introduction

**Figure 1:**
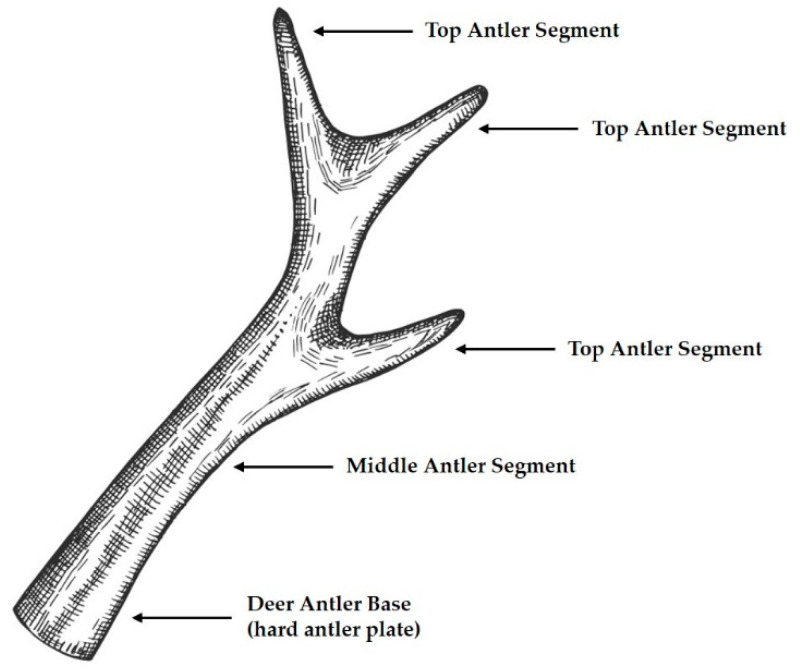
Parts of Deer antler.

In recent years, people have become increasingly aware of the importance of diet in health, which has contributed to the popularity of nutritional supplements among consumers. Deer antler has been used as a health food and medicine in China, Japan, and Korea for thousands of years. As the only fully regenerable mammalian organ, deer antler has generated interest as an animal-based medicine that can be obtained without harming the animal [1]. Traditional medicine practitioners believe that deer antler can strengthen bones, treat nervous disorders, activate blood circulation, and replenish vital energy [2]. Antlers are divided into three parts according to the degree of ossification: Top Antler Segment, Middle Antler Segment, and Deer Antler Base (hard antler plate) (Figure 1). Recent studies have isolated various of bioactive compounds from deer antler, such as peptides, lipids, polysaccharides, proteins, nucleotides, glycoproteins, and trace elements [3]. Several studies have been conducted using molecular techniques and cellular and animal models to confirm the pharmacological effects of these components. Deer antler has great potential in treating many diseases such as bone injuries [4], neurodegenerative diseases [5], tumors [6], and inflammatory conditions [7].

Bioactive peptides are composed of more than two amino acid residues and have low molecular weights (usually no more than 20 amino acid residues), which can be consumed for various activities in the body [8]. The composition of amino acids and the peptide sequence allow different bioactive peptides to have different biological activities. Bioactive peptides usually exert higher levels of bioactivity than whole proteins due to more functional active groups are present in the former than the latter [9]. Because of the superior functional activity, bioactive peptides are believed to have health-promoting abilities. Bioactive peptides are usually obtained from intact proteins, and deer antler, which contains about 50% of the dry weight in protein (Table 1) [10], is considered a good source of bioactive peptides. DAPs possess antioxidant [11], anti-inflammatory [12], hypoglycemic [13], anti-organ fibrosis [14], anti-aging [15], anti-tumor [16], anti-neurological disease [17], and bone regeneration-promoting properties [18].

Many studies have focused on DAPs, particularly on their roles in disease treatment and health maintenance. Therefore, this review describes the current common extraction methods of DAPs and presents the health effects as well as the therapeutic potential of DAPs. The disease therapeutic capabilities of DAPs are emphasized, with particular attention to the molecular mechanisms described. The limitations facing the extraction and application of DAPs are also briefly discussed to guide their further research.

## 2. Extraction of DAPs

The intact proteins in the organism are in an inactive state due to the conformational arrangement of proteins that hide hydrophobic and reactive groups deep in the protein structure. Thus, bioactive peptides must be extracted using specific methods by some means [22]. Currently, the methods used for the extraction of DAPs include water extraction, enzymatic hydrolysis, chemical hydrolysis, organic solvent extraction, fermentation extraction, and ultrasonic assisted extraction (Table 2). It is worth noting that deer antler was previously used as a traditional medicine through water extraction, therefore many studies on DAPs up to now still use this process with improvements.

The water extraction is easy to perform, but the extremely low recovery of bioactive components limits the study of DAPs in water extracts. This limitation can be attributed to the difficulty of disrupting protein folding when water is used as an extraction medium [57]. Moreover, the composition of water extracts is very complex, which affects more in-depth studies. Therefore, studies on obtaining DAPs by using different methods are gaining attention.

Deer antler is rich in collagen [58], and the use of acid as an extraction medium can open the cross-linkage bonds between collagen molecules, while disrupting tryptophan, serine and tyrosine, allowing intact proteins to be cleaved into peptide segments. Acetic acid with pH between 3.5–4.0 is widely used for DAPs extraction. Shu-Wen et al. used acetic acid to extract DAPs with cell proliferation-promoting activity [46], and Chaohua et al. found that acetic acid-extracted DAPs have positive implications for neurodegenerative diseases [47]. In addition, acid-extracted DAPs have been found to be useful in the treatment of tumors and heart muscle damage, as well as in lowering blood sugar [13,48,50].

In recent years, enzymatic hydrolysis has been considered effective in obtaining bioactive peptides from proteins. At present, Alcalase, Protamex, Pepsin, and Trypsin have been reported to be used to extract DAPs. The active sites of enzymes make their differences in substrate–enzyme interaction, which results in the variation of different enzymolysis products. For instance, Alcalase was preferential in cleaving the end peptide bonds of uncharged residues (Leu, Glu, Met, Lys, Tyr, and Gln) [59], whereas Pepsin and Trypsin are preferred the hydrophobic/aromatic residues of Tyr, Ile, Met, Val, and Leu as well as the specific residues of Arg and Lys at the *C*-terminal [60]. Therefore, the selection of different enzymes and extraction protocols can yield DAPs with different biological functions. DAPs extracted with Alcalase have strong antioxidant and anti-inflammatory activities [38,39]. Peptides obtained from the hydrolysis of deer antler by Protamex have the ability to inhibit adipogenesis and alleviate fatness [40]. Ultrasound-assisted pepsin-extracted DAPs can promote osteoblast proliferation and differentiation [46]. The DAPs obtained after pepsin and trypsin simulated gastrointestinal digestion function in antioxidation, anti-inflammation, cell proliferation promotion and neurodegenerative disease alleviation [42,43,45].

In addition to traditional water extraction, acid extraction, and enzymatic hydrolysis, studies on the extraction of DAPs using buffer solution or fermentation have been reported. DAPs extracted from defatted deer antlers using NaCl-HCl buffer can accelerate hair growth [51]. DAPs obtained by fermentation using *Bacillus subtilis* exhibited hematopoietic effects and showed improvement in hemolytic anemia [56]. Different extraction methods have been used to bring many interesting biological activities to DAPs, which means the extraction and application of DAPs have attracted the attention of researchers. However, the extraction and separation of DAPs are mostly at the crude extract stage, which somehow limits the in-depth study of DAPs. Therefore, research on the extraction and preparation of DAPs should focus on the development of novel extraction protocols and separation protocols.

## 3. Biological Functions of DAPs

### 3.1. Antioxidant Activity

Oxidative damage is closely related to the accumulation of free radicals and reactive oxygen species (ROS) [61]. Due to environmental factors and unhealthy lifestyles, excessive levels of ROS and free radicals may accumulate in the body, leading to redox imbalance and causing oxidative damage to the organism. The disruption of biomolecular structures in the body brought about by oxidative damage is significant for the development of disease, and common chronic diseases such as gastrointestinal inflammation, heart disease, and neurodegenerative diseases are closely associated with oxidative damage [62]. Therefore, the scavenging of ROS in the metabolic system by natural antioxidants and the prevention of oxidative damage have been widely investigated. Some current studies suggest that the antioxidant activity of peptides is related to amino acid composition. The amino acid residues associated with antioxidant activity are mainly found in hydrophobic and aromatic amino acids because they serve as hydrogen donors to transfer electrons for scavenging free radicals [63]. In addition, the amino acid residues with metal chelating ability can scavenge ferrous ions to inhibit oxidation reactions. Deer antler is rich in Glu, Pro, Asp, Gly, Arg, etc., and can be considered as a high-quality source of antioxidant peptides (Table 3). For example, DAPs obtained from deer antler gelatin hydrolysis had the highest percentage of Gly, Ala, and Pro, thus showing 1,1-Diphenyl-2-picrylhydrazyl radical 2,2-Diphenyl-1-(2,4,6-trinitrophenyl)hydrazyl (DPPH) radical scavenging activity, Ferric ion reducing antioxidant power (FRAP) radical scavenging activity, and 2, 2’-azino-bis(3-ethylbenzothiazoline-6-sulfonic acid) (ABTS) radical scavenging rate of 94.51% [11]. The tetrapeptide TAVL obtained by hydrolysis of deer antler using Alcalase shows strong peroxyl radical scavenging activity (IC50 = 51.16 μM) because of the high content of hydrophobic amino acids [38]. Meanwhile, DAPs have good thermal and emulsion stability, which can be applied in the food, pharmaceutical, and cosmetic industries [11].

The establishment of cellular and in vivo experiments provides an intuitive model to study the biological mechanisms of antioxidant activity of DAPs, which are usually inextricably linked to the inhibition of ROS and regulating oxidation-related and apoptosis-related factors. In the H_2_O_2_-induced Human Umbilical Vein Endothelial Cells (HUVEC) injury model, DAPs reduce ROS-induced cell injury and apoptosis by blocking the Caspase-3 signaling pathway, upregulating the expression of superoxide dismutase (SOD) and glutathione (GSH) and inhibiting the increase of intracellular Malondialdehyde (MDA) levels [49]. The Caspase-3 signaling pathway plays key roles in apoptosis and pyroptosis, in which Caspase-3 activation and c-Jun *N*-terminal kinase (JNK) phosphorylation are closely associated with oxidative damage and trigger pyroptosis [64]. In SH-SY5Y human neuroblastoma cells, DAPs exhibit antioxidant activity by downregulating the levels of Caspase-12 and p-JNK [17]. Metallothionein (Mt)2, Mt1, Sod3, NDUFA4 mitochondrial complex associated like 2(Ndufa4l2), hypoxia inducible factor 1 α(Hif1α), Sod2, NAD(P)H quinone dehydrogenase 1(Nqo1), glutathione-disulfide reductase (Gsr), and nuclear factor kappa B 1(NF-κB 1) were all found to be involved in the oxidative stress response and play important roles in scavenging free radicals, maintaining redox reactions, and regulating mitochondrial respiration. In primary chondrocytes, DAPs significantly upregulated the expression of these factors, suggesting the role of DAPs in enhancing cellular antioxidant capacity [26]. 2,2′-azobis(2-methylpropionamidine) dihydrochloride(AAPH)-induced oxidative stress may lead to elevated ROS, lipid peroxidation, and cell death. In the Human hepatocyte-derived cell model of AAPH-induced injury, DAPs resist ROS elevation and cellular damage [38]. In addition, DAPs inhibited cell death, ROS production, and lipid peroxidation in Zebrafish larvae [38]. Both cellular and in vivo experiments demonstrated the resistance of DAPs to oxidative damage. Administration of DAPs reduced cell damage and downregulate Phosphoinositide-3 kinase (PI3K) expression in Human hepatoellular carcinomas 2 (HepG2) and SMMC7721 cells as well as mouse liver tissue [62]. PI3K has been found to be closely associated with apoptosis caused by oxidative stress [65]. In diabetic mice, the levels of antioxidant enzymes (SOD, CAT) and Total antioxidant capacity (T-AOC) in the liver and serum increased while MDA levels decreased after the administration of DAPs. This result suggests that DAPs may exhibit antioxidant activity by acting on enzymatic and non-enzymatic antioxidants [51].

### 3.2. Anti-Inflammation

Inflammation is a complex physiological response of the body to fight against viral invasion, microbial infection, and cellular damage. However, excessive and prolonged inflammation causes damage to tissues and organs and may lead to many acute and chronic diseases [66]. Suppression of excessive and uncontrolled inflammation is important to prevent inflammatory diseases. Downregulation of the expression of these factors can modulate or suppress excessive or persistent inflammation. As reported in previous studies, DAPs can regulate inflammation in cellular or animal assays (Table 4). For example, treatment of rats with streptococcal cell wall (SCW)-induced arthritis by DAPs revealed that inflammation-induced leukocytosis was suppressed and adhesion of blood cells to the endothelium was reduced, which facilitated a reduction in the recruitment of inflammatory cells to the joints and thus modulated arthritis [67]. Similarly, in vivo administration of DAPs to rats with type II collagen-induced arthritis can inhibit the onset and progression of arthritis. Furthermore, the inflammatory response is characterized by elevated levels of pro-inflammatory cytokines, such as interleukin (IL)-1, IL-6, tumor necrosis factor (TNF)-α and interferon (IFN)-γ [68], and consequently by the development of disease. Lymph node cells were isolated from model rats and administered with DAPs, and it was found that the expression levels of IL-1β, IL-2, IL-6, TNF-α and IFN-γ were significantly reduced [67,69], while the acceleration of arthritis severity could be attributed to the elevation of these factors [70]. This demonstrates the modulatory effect of DAPs on T-cell-mediated immune responses. Treatment with DAPs can effectively suppress LPS-induced inflammation in nucleus pulposus cells; decrease the levels of pro-inflammatory cytokines IL-1β, IL-6, and TNFα; and reduce the viability of MDA [12]. Assays for oxidative stress and inflammation-related proteins showed that DAPs reversed the LPS-induced elevation of p-NF-κBp65, phosphonated inhibitor of nuclear factor kappaB (p-IκB)α, p-Jnk, phosphonated inhibitor of extracellular regulated protein kinases (p-Erk), and p-P38 levels, and this effect can be inhibited by the p38 mitogen-activated protein kinase (MAPK) inhibitor SB203580 [12,45]. This result suggests that the anti-inflammatory ability of DAPs can be achieved via the MAPK/NF-κB pathway. In addition to the MAPK/NF-κB pathway, the anti-inflammatory mechanism of DAPs may also be related to the inhibition of the Rho/NF-κB pathway. After the administration of LPS to mice to produce lung injury, the myeloperoxidase (MPO) content of the lungs was significantly increased, which revealed the active state of neutrophils, but MPO activity decreased after treatment with DAPs. The immunoblot analysis confirmed that DAP administration can inhibit the levels of Rho and its downstream molecules Rho-associated protein kinase (ROCK)-I and ROCK-II, as well as the phosphorylation of NF-κB and IκBα, suggesting the inhibitory effect of DAPs on the Rho/NF-κB pathway [71]. During bone formation and osteoblast proliferation and differentiation, Epidermal Growth Factor (EGF)/EGFR is involved in the regulation of bone homeostasis [72]. Moreover, EGF/EGFR controls inflammation in osteoblasts by regulating factors such as MDA, IL-1β, IL-6 and TNFα [73]. Treatment of MC3T3-E1 cells with DAPs can significantly upregulate the expression of EGF, EGFR, NRF-2 and HO-1. However, the anti-inflammatory effect of DAPs was almost abolished after EGF knockdown using siRNA [18]. This phenomenon suggests that the anti-inflammatory activity of DAPs in osteoblasts is related to the EGF/EGFR pathway.

A previous study used RNA-seq technology to analyze the glabellar cartilage of rats treated with DAPs and found that DAPs treatment downregulated the expression of 30 inflammation-associated Differentially Expressed Genes (DEGs) [74]. Another study reported RNA-seq results before and after treatment of mouse primary chondrocytes with DAPs. This study found that the expression of anti-inflammatory regulators, such as Protein Tyrosine Phosphatase Non-Receptor Type 2 (Ptpn2), avian reticuloendotheliosis viral (v-rel) oncogene related B (Relb), sphingosine-1-phosphate receptor 3 (S1pr3), peroxisome proliferator activated receptor delta (Ppard), selectin P (Selp), and adenosine A1 (Adora1) was upregulated after DAPs treatment. These results indicates that DAPs treatment exerts effective anti-inflammatory activity in chondrocytes [26]. Enzymatic hydrolysis likewise brought inflammatory regulatory activity to the DAPs. The peptide obtained from Alcalase treatment of deer antler can inhibit of NO production in RAW264.7 cells and zebrafish, and immunoblotting confirmed that this effect could be attributed to inhibition of myo-inositol-1-phosphate synthase (iNOS) and cytochrome c oxidase subunit II (COX-2) expression [39]. Four peptides (VH, LAN, AL, IA) identified from the pepsin and trypsin hydrolysis products all had inflammation-modulating activity and inhibited intracellular NO production. However, the NO inhibitory activity of the peptide mixture was higher than that of any of the purified peptides, suggesting the synergistic effect of DAPs in inflammatory resistance [45].

### 3.3. Effect on Bone and Cartilage

Bone is a dynamic vascularized living tissue that provides structure and support for the body and stores minerals, such as calcium and phosphorus [75]. Bone remodeling maintains normal bone form. Osteoblasts and osteoclasts are the two main bone cells involved in bone remodeling, and the cellular activity between them is a key factor in maintaining the balance among bone resorption and bone formation [76]. The imbalance in the bone formation and bone resorption leads to bone loss and consequently to diseases, such as osteoporosis and femoral head necrosis [77]. Several studies have shown that DAPs promote osteogenesis and inhibit the development of osteoporosis (Table 5). For example, Zhang et al. treated rats with DAPs after the removal of ovaries causing osteoporosis and found that osteoporosis symptoms were relieved and Bone Water Concentration (BWC), Bone Mineral Content (BMC), Bone Mineral Density (BMD), calcium ion, and phosphorus levels were increased. Paraffin sections showed that the trabecular network was restored, and the number and volume of trabeculae increased significantly, thus representing an increase in bone strength. In addition, decreased levels of IL-1 and IL-6 were detected in several chondrocyte and osteoblast-like cells, inhibiting osteoclast differentiation and osteoclast formation [78]. Experiments on cellular models likewise confirm this opinion. Primary osteoblasts tend to differentiate into osteoblasts, and TNF-α can inhibit this activity. After the addition of DAPs, the osteogenic differentiation of primary osteoblasts was promoted, and the inhibitory effect of TNF-α was suppressed. In addition, the expression levels of transcription factor Runx2 and osteogenic-specific genes alkaline phosphatase (ALP), osteocalcin (OCN), black spleen (BSP), and secreted phosphoprotein (OPN) were significantly elevated, and the accumulation of NF-κBp65 was inhibited, thus representing that DAPs promoted osteogenic differentiation and inhibited osteolytic differentiation of downregulating NF-κB/p65 pathway [79]. Studies on bone marrow mesenchymal stem cells showed that DAPs activated the BMP-2/Smad1,5/Runx2 pathway, increased extracellular matrix mineralization, and promoted the proliferation and differentiation of osteoblasts [23]. The impaired insulin signaling caused by diabetes has been shown to cause osteoporosis, and the phosphorylation levels of InsR, IRS-1, and IRS-1, AKT serine/threonine kinase 1 (AKT), and ERK were significantly increased after DAPs treatment, suggesting that DAPs can promote osteoblast proliferation through regulating the insulin signaling pathway to treat diabetes-induced osteoporosis [80]. Serum proteomic analysis of rats in the osteoporosis model suggested that DAPs upregulate 23 proteins that promote bone formation, including B2m and IL-16, and downregulate 10 proteins that may inhibit bone formation from affecting the dynamic balance between osteoblasts and osteoclasts [25].

DAPs have also been shown to positively affect arthritis because of the inflammatory modulating ability and the promotion of bone formation. In both rats and mice, the therapeutic effect of DAPs on arthritis has been demonstrated [24,67]. The therapeutic effect of DAPs on arthritis may be attributed to the inhibition of inflammation and modulation of the extracellular matrix, as well as blocking the recruitment of inflammatory cells at the joint. DAPs were found to promote the TK signaling pathway and thus chondrocyte proliferation in primary chondrocytes [81]. Furthermore, RNA-Seq analysis of primary chondrocytes revealed that DAPs regulate a variety of transcription factors related to proliferation, differentiation, anti-inflammation, and immune regulation, as well as growth factors and morphogens, to achieve the goal of promoting chondrocyte proliferation and resisting inflammation [26]. Proteomic analysis identified 192 upregulated proteins, of which all differentially expressed proteins related to intracellular transport, secretion, chromatin structure, and cytoskeleton were expressed at elevated levels. In particular, the expression levels of the cell proliferation markers Mki67 and STMN1, the differentiation inhibitor ACP5, the apoptosis inhibitor Ndufa4l2 and Rcn1 were significantly increased, suggesting that DAPs promote chondrocyte proliferation and inhibit apoptosis through multiple cellular processes such as protein synthesis, ribosome formation and cytoskeleton reorganization [26]. RNA-Seq analysis of rat xiphoid cartilage identified 892 DEGs, of which 181 were upregulated genes and 711 were downregulated genes. Among them, the gene expression levels of cartilage growth and regeneration were significantly increased, and those related to cartilage formation and cell proliferation were similarly elevated. In contrast, the downregulated genes were mainly concentrated in inflammation-related genes [74]. The therapeutic effects of DAPs on arthritis have been demonstrated in a series of studies in rat and mouse models, respectively. In a rat model of arthritis induced by type II collagen and treated with DAPs, IL-1β, IL-2, IL-6, TNF-α, IFN-γ, and dihydroorotate dehydrogenase (DHO-DHase) were inhibited [69,82]. Treatment with DAPs in a mouse arthritis model resulted in a significant decrease in TNF-α and neutral endopeptidase activity and relief of arthritis symptoms [83]. DAPs can inhibit peptidoglycan-polysaccharide fragments-induced joint swelling, deformation, and progression of chronic arthritis in rats with polyarthritis, confirming that DAPs can be used as a therapeutic option for acute and chronic arthritis [67].

### 3.4. Effects on Neurological Diseases

The development of neurodegenerative diseases is influenced by excessive and uncontrolled inflammation, and the imbalance of intracellular redox homeostasis [84]. Neuroinflammation leads to microglia activation and release of multiple inflammatory mediators such as pro- and anti-inflammatory cytokines and neurotoxic mediators [85,86]. Oxidative stress causes free radicals to attack nerve cells [87], leading to catastrophic neurodegeneration, and exacerbates the production and aggregation of β-amyloid and phosphorylation of tau proteins [88,89], which predisposes to a vicious pathogenic cycle of neurodegenerative diseases. DAPs have received attention in the treatment of neurodegeneration because of their anti-inflammatory and antioxidant activities (Table 6). DAPs treatment to human neuroblastoma cells with H_2_O_2_-induced injury significantly reduced apoptosis, BCL2 associated X (Bax) and Caspase-3 expression levels were inhibited, while B cell leukemia/lymphoma 2 (Bcl2) expression was promoted [90]. Bax and Caspase-3 take important roles in apoptosis [33], and Bcl2 has been shown to inhibit cytotoxin-induced cell death [91]. In another study using sevoflurane to mediate neuronal cell injury, DAPs can modulate the expression of of Bax, Caspase-3, and Bcl2 expression levels and inhibition of apoptosis [92]. In vivo experiments using mice confirmed DAPs can also restore the number of hippocampal neurons in the brain and reduce intracerebral damage by downregulating the expression of corticotropin releasing hormone (CRH), adreno cortico tropic hormone (ACTH), Corticosterone (CORT), Recombinant Glucocorticoid Receptor (GR), Mineralocorticoid receptor (MR) and maintaining the homeostasis of the Hypothalamic-Pituitary-Adrenal (HPA) axis [90]. Two in vivo studies on morphine found that treatment of mice with DAPs can inhibit morphine-induced somatic dependence, reverse tolerance, presynaptic dopamine (DA) receptor dysfunction, and postsynaptic DA receptor hypersensitivity and avoid dopamine depletion [33,34], one of the pathological features of Parkinson’s disease [93]. Overall, DAPs indicated strong neuroprotective ability. In addition, DAPs effectively alleviated the collapse of mitochondrial membrane potential, endoplasmic reticulum stress, and elevated ROS in neuronal cells and also inhibited the activation of the Ca^2+^-calpain-caspase-12 pathway [17]. This result suggests the potential therapeutic ability of DAPs in neurodegenerative diseases, especially Parkinson’s disease. Inactivation of tyrosine hydroxylase is closely associated with degeneration of the substantia nigra state and is a classic motor feature of Parkinson’s disease [94]. The survival of tyrosine hydroxylase-positive neurons is one of the critical signatures in the development of Parkinson’s disease. In an in vivo experiment, using deer antler extract to treat a rat model of Parkinson’s disease, the death of tyrosine hydroxylase-positive neurons was significantly inhibited [47]. In addition, the decreased levels of γ-aminobutyric acid (GABA) and Glu reflected the restricted development of Parkinson’s disease, while the increased levels of GAP-43 and NF-H reflected the promotion of neuronal growth and plasticity [47]. Alzheimer’s disease is another common neurodegenerative disease, and abnormal aggregation of β-amyloid is often considered as being the main cause of Alzheimer’s disease development [95]. Treatment with DAPs extracted in three different ways in the Alzheimer’s disease model of *C. elegans* significantly improved motility and inhibited β-amyloid deposition in *C. elegans*, with the DAPs extracted using combined pepsin and trypsin hydrolysis having the best efficacy [43]. Oxidative stress also plays important roles in the development of Alzheimer’s disease. DAPs have been indicated to activate the antioxidant signaling pathway in *C. elegans* and upregulate Protein skinhead-1 (SKN-1), heat shock transcription factor 1 (HSF-1), Fork-head domain-containing protein (DAF-16), SOD-3 [43], which have been reported to have critical effects in regulating β-amyloid toxicity [96,97].

Stroke is the fourth leading cause of death worldwide and is highly disabling [100]. About 80% of strokes are ischemic in nature and are primarily associated with cerebral ischemia/reperfusion injury, oxidative stress, and inflammation [101]. Increased glutamate levels and increased intracellular calcium levels have been reported after cerebral ischemia [102]. DAPs treatment reduces intracellular Ca^2+^ levels in human neuroblastoma cells, suggesting the potential therapeutic ability for stroke [17]. DAPs also demonstrated therapeutic potential for stroke in vivo in animals. Administration of DAPs to rats treated with cerebral artery occlusion can significantly reduce infarct volume, neurological recovery, and inflammation-related factors, such as IL-1β, IL-6, and TNFα, and significantly upregulate endogenous antioxidant proteins such as nuclear factor, erythroid derived 2 (Nrf-2), HO-1, and inflammation regulators p-component of inhibitor of nuclear factor kappa B kinase complex (IKK)α, p-IKKβ, p-NF-κBp65, and p-IκBα [99]. Another study also observed that DAPs can downregulate the expression of oxidative and inflammatory-related genes [98]. DAPs can also significantly upregulate the expression of glial cell derived neurotrophic factor (GDNF) and nerve growth factor NGF, suggesting the neuroprotective effect of DAPs after stroke [103,104]. Pyroptosis is a pro-inflammatory programmed cell death induced by NLRP3 inflammatory vesicles [105], and its induced neurological dysfunction can exacerbate depression development [106]. DAPs are considered as potential therapeutic options for depression because of their protection of neuronal cells and inhibition of inflammation and oxidative stress. Treatment with DAPs after the induction of depression in a mouse model can significantly reduce depression-like behaviors, lead to a significant reduction in neuronal damage, modulate the AMPK/Sirt1/NF-κB/NLRP3 pathway, and inhibit its mediated pyroptosis [54], thus indicating that DAPs are a candidate treatment for depression. However, further studies confirming other positive effects of DAPs in individuals with depression are lacking.

### 3.5. Other Physiological Regulatory Activities

DAPs exert anti-cancer activities. In particular, peptides extracted from Hard antler plates inhibit the proliferation of breast cancer cells by arresting the cell cycle and inhibiting telomerase activity [50]. DAPs also inhibit the invasion of breast and prostate cancer cells [55,107]. In another in vivo study, DAPs administered by gavage inhibit breast cancer in mice [16]. DAPs have also been shown to rescue acute liver injury through MAPK and NF-κB signaling pathways [108]. Excessive accumulation of extracellular matrix components, especially collagen, is an important etiology of cardiac and hepatic fibrosis [109], and treatment with DAPs significantly inhibits hepatic collagen deposition in mice and induces liver fibrosis via the TGF-β/Smad pathway [44]. In addition, for myocardial fibrosis and cardiomyocyte apoptosis, DAPs showed positive effects through similar mechanisms [46,110]. The water extract of deer antler enhances the beating capacity of the heart and may be very beneficial in enhancing heart activity [111]. The role of DAPs in the treatment of pulmonary fibrosis may be related to the ROCK/NF-κB signaling pathway [14]. DAPs extracted by different methods have immunomodulatory effects and regulatory effects on the expression Th1 and Th2 cytokines [112,113]. In addition, a glycine- and proline-rich peptide isolated from a deer antler exhibited good glucose metabolism-promoting activity and has been suggested to be applied to treat diabetes [13,51]. DAPs promote myogenin differentiation 1 (MyoD1), myogenic factor 5 (Myf5), and myogenin in a C2C12 cell model and inhibit muscle atrophy induced by senescence [114]. A study found that deer antler extract promoted hair follicle growth. Further in-human clinical trials confirmed that deer antler extract can promote hair growth without irritating the head skin, implying that deer antler extract can be used as a mild hair growth drug to treat hair loss [115].

## 4. Conclusions

Natural products have been used for healing in Far Eastern countries for thousands of years. In China, treatment with preparations from animals has an important place in traditional medicine. Deer antler has attracted attention because of its repeated regeneration and fast growth rate, and it has been consumed as medicine and as a health food for thousands of years. Regular consumption of deer antler extract provides vitality, strengthens bones, and has positive effects on the treatment of many diseases. Studies show that DAPs, as an active ingredient of deer antler, may contribute to the conventional treatment of many diseases owing to their pharmacological properties. Intensive studies on the mechanism of action of DAPs may have positive implications for the development of effective natural medicines. However, supplementation of natural extracts also carries the potential risk of side effects and interaction with other drugs. It is also necessary to establish uniform conditions for the production of specific peptides, to elucidate the relationship between amino acid composition, peptide structure and bioactivity, and to conduct more extensive experiments to determine the linkages and differences between the functions of DAPs obtained by different extraction methods. In addition, DAPs are commonly administered by oral ingestion. However, few studies have focused on the digestive stability and absorption patterns of DAPs after ingestion, so more studies on the absorption as well as utilization of DAPs are needed to determine their bioavailability and stability in vivo. In recent years our understanding of deer antler as a health food and source of bioactive substances has evolved. However, although there are many promising data, further studies in extraction and clinical aspects are needed to evaluate the therapeutic benefits of DAPs.

## Figures and Tables

**Table 1:** Chemical compositions of different sections of Deer antler.

Item	Top Antler Segment	Middle Antler Segment	Deer Antler Base (Hard Antler Plate)	References
Ash (%)	50.3	48.4	46.1	[19]
Amino acid (%)	46.4	49.0	44.6	[20]
Calcium (%)	19.7–20.5	21.1–27.7	19.6–21.2	[21]
Phosphorus (%)	9.7–11.3	13.0	10.0–12.3	[21]

**Table 2:** Extraction method and biological activity of DAPs.

Extracting Media	Biological Activities	Origin	References
Water	Anti-osteoporotic activity	Deer antler	[23]
Water	Anti-cancer activity	Ultrafine lyophilized powder of deer antler	[16]
Water extraction assisted by ultrasound	Anti-arthritic activity	Red deer antler	[24]
Cold water	Activity in regulating bone formation	Deer antler	[25]
Cold water	Antioxidant activity, anti-inflammatory activity, immunomodulatory activity	Deer antler	[26]
Cold water	Cell proliferation promoting activity, promotion of bone formation	Deer antler	[27]
Cold water	Anti-cancer activity	Deer antler base	[6]
Cold water	Hair growth promoting activity	Top Antler Segment	[28]
Cold water extraction assisted by ultrasound	Intestinal cell barrier protection activity	Deer antler	[29]
Hot water	Anti-bone damage activity	Deer antler	[30]
Hot water	Antioxidant activity, reduce liver damage	Top Antler Segment	[31]
Hot water	Anti-invasive, anti-inflammation activity	Deer antler	[32]
Hot water	Antidopaminergic	Deer antler	[33]
Hot water	Neuroprotective activity	Deer antler	[34]
Hot water	Anti-aging activity	Deer antler	[35]
Hot water	Anti-inflammation activity, against lung damage avtivity	Dry powder of velvet antler	[36]
Hot water	Anti-cancer activity	Top Antler Segment	[37]
Alcalase solution (pH = 8)	Antioxidant activity	Deer antler	[38]
Alcalase solution (pH = 8)	Antioxidant activity, anti-inflammatory activity	Dry powder of velvet antler	[39]
Protamex solution (pH = 6)	Inhibits fat production activity	Dried deer antler powder	[40]
Pepsin hydrolysis assisted by ultrasound	Cell proliferation promoting activity	Deer antler solid glue	[41]
Pepsin solution (pH = 2) Trypsin solution (pH = 7.8–8.5)	Inhibits scar formation	Deer antler	[42]
(1) Pepsin solution (pH = 2) (2) Trypsin solution (pH = 7.8–8.5)	Neuroprotective activity, antioxidant activity	Traditionally-dried two-branched deer antler	[43]
Hot water Trypsin solution (pH = 8)	Antioxidant activity	Deer antler base	[11]
(1) Na2HPO4-NaOH buffer (pH = 12, 50 mmol/L EDTA,0.5 mol/L NaCl) (2) Trypsin solution (pH = 9)	Against liver toxicity	Cornu Corvi nippon parvum	[44]
100 mM Tris, 6M guanidine-HCl, 20mM EDTA-2Na Pepsin solution (pH = 2) Trypsin solution (pH = 6.8)	Anti-inflammation activity	Deer antler	[45]
Ice-cold acetic acid solution (pH = 3.5)	Antioxidant activity, neuroprotective activity	Deer antler	[13]
Ice-cold acetic acid solution (pH = 3.5)	Hypoglycemic activity	Sika antler powder	[46]
Ice-cold acetic acid solution (pH = 3.5)	Cell proliferation promoting activity	Dried antler	[47]
Ice-cold acetic acid solution (pH = 4)	Anti-Parkinsonian activity	Deer antler	[48]
Ice-cold acetic acid solution (pH = 4)	Anti-fibrotic, anti-apoptotic, cardioprotective effect	Deer antler	[49]
Ice-cold acetic acid solution (pH = 4)	Antioxidant activity, anti-apoptotic activity	Deer antler	[50]
Pre-cooled acetic acid solution	Anti-cancer activity	Hard antler plate	[51]
50% ethanol Ice-cold acetic acid solution (pH = 3.5)	Antioxidant activity, hypoglycemic activity, hypolipidemic activity	Dried antler	[52]
HAc-NaAc buffer (pH = 3.5)	Promotes wound healing, proliferative activity	Deer antler	[53]
Acetone and chloroform-methanol mixture0.02 M NaCl-HCl buffer (pH = 6)	Promotes wound healing, promotes hair growth	Red deer antler	[54]
Cold water Formic acid solution (0.2%)	Antidepressants	Deer antler	[55]
70% ethanol	Anti-cancer activity	Top Antler Segment	[56]
B. subtilis KH-15	Improve hemolytic anemia	Dried antler	[57]

**Table 3:** Antioxidant activity of DAPs.

Origin	Extracting Media	Antioxidant Activity	References
Deer Antler	Ice-cold acetic acid solution (pH = 4)	HUVEC cell: Mitigation of H2O2-induced cytotoxicity and apoptosis. Blocking Caspase-3 signaling pathway. ROS, MDA↓; SOD, GSH↑	[49]
Deer Antler Base	(1) Hot water (2) Trypsin solution (pH = 8)	In vitro: DPPH, ABTS, FRAP Radical Scavenging Activities	[11]
Deer Antler	Cold water	Primary chondrocytes: Mt2, Mt1, Sod3, Ndufa4l2, Hif1α, Sod2, Nqo1, Gsr, and Nfkb1↑	[26]
Top Antler Segment	Hot water	HepG2 and SMMC7721 cells: Inhibition of AAPH-induced cell death. PI3K↓	[31]
		In vivo (male C57BL/6J mice): Inhibition of lithocholic acid-induced oxidative stress in the liver: ROS, MDA, OGG1↓; SOD↑	
Deer antler	Alcalase solution (pH = 8)	In vitro: Peroxyl Radical Scavenging Activities	[38]
		Human hepatocyte-derived cell: Mitigation of AAPH-induced cytotoxicity. ROS↓	
		In vivo (Zebrafish): Inhibition of AAPH-induced cell death, reactive oxygen species production and lipid peroxidation	
Deer antler	Ice-cold acetic acid solution (pH = 3.5)	SH-SY5Y human neuroblastoma cell: Caspase-12, p-JNK ↓	[17]
Dried antler	(1) 50% ethanol (2) Ice-cold acetic acid solution (pH = 3.5)	In vivo (C57BL/6J mice): Inhibition of oxidation levels in liver and serum of diabetic mice: SOD, CAT, T-AOC↑; MDA↓	[51]

**Table 4:** Anti-Inflammation activity of DAPs.

Origin	Extracting Media	Antioxidant Activity	References
Deer Antler	Hot water	In vivo (Rats): Inhibition of SCW-induced leukocytosis, decrease blood cell adhesion to the endothelium	[67]
Deer antler	Water	Lymph node cells: IL-1β, IL-2, IL-6, TNF-α, IFN-γ↓	[69]
		In vivo (Rats): Inhibit the development and progression of arthritis	
Deer antler	Hot water	Nucleus pulposus cells: MDA, IL-1β, IL-6, TNFα, p-NF-κBp65, p-IκBα, p-Jnk, p-Erk, p-P38↓	[12]
Deer antler	Hot water	In vivo (mice): Reduction of acute lung injury, reduced lung wet/dry weight ratio. MPO, MDA, IL-1β, IL-6, TNFα, phosphorylations of NF-κB, IκBα, the expression of Rho, ROCK-I, ROCK-II↓; SOD↑	[71]
Deer Antler	Hot water	MC3T3-E1: IL-1β, IL-6, TNFα, NF-κBp65, IκBα↓; EGF, EGFR, Nrf-2, HO-1↑	[18]
Deer Antler	Cold water	In vivo (Rats): 30 inflammation-associated genes significantly downregulated	[74]
Deer antler	Cold water	Primary chondrocytes: Ptpn2, Relb, S1pr3, Ppard, Selp and Adora1↑	[26]
Dry powder of velvet antler	Alcalase solution (pH = 8)	RAW264.7 cells: NO inhibitory activities in LPS-induced cells, iNOS, COX-1↓	[39]
		In vivo (Zebrafish): ROS, NO↓	
Deer Antler	(1) 100mM Tris, 6 M guanidine-HCl, 20 mM EDTA-2Na (2) Pepsin solution (pH = 2) (3) Trypsin solution (pH = 6.8)	RAW264.7 cells: NO inhibitory activities in LPS-induced cells	[45]

**Table 5:** Effect on bone and cartilage of DAPs.

Origin	Extracting Media	Bioactivities	References
Effects on osteoporosis			
Deer antler		In vivo (Rats): Restoration of bone trabecular network; BWC, BMC, BMD, Ca^2+^, phosphorus↑	[78]
		Rabbit costal cartilage cells, human fetal articular cartilage cells, and chicken fetal osteoblast-like cells: IL-1, IL-6↓	
Deer Antler		Primary osteoblastic cells: Promotes osteogenic differentiation and inhibits osteolytic differentiation. Runx2, ALP, OCN, BSP, OPN↑, NF-κBp65↓	[79]
Deer antler solid glue	Pepsin hydrolysis assisted by ultrasound	Bone marrow mesenchymal stem cells: Promotes proliferation and osteogenic differentiation; BMP7↑	[41]
Deer Antler	Water	Bone marrow mesenchymal stem cells: Activation of BMP-2/Smad1,5/Runx2 pathway; extracellular matrix mineralization, ALP↑	[23]
Deer Antler		MC3T3-E1: InsR, IRS-1, p-InsR, p-IRS-1, p-AKT, p-ERK↑	[80]
Deer antler solid glue	Pepsin hydrolysis assisted by ultrasound	In vivo (Rats): 23 upregulated genes, 10 downregulated genes that regulate cytoskeletal organization, immunity and inflammation to control bone formation and remodeling	[25]
Effects on femoral head necrosis			
Deer antler	Hot water	Primary osteoblastic cells: Regulates cell cycle and promotes cell proliferation. Alkaline phosphatase↑	[30]
		In vivo (Rats): Inhibits femoral head cell necrosis. Hydroxyproline↓, aminohexose↑	
Effects on arthritis			
Red deer antler	Water extraction assisted by ultrasound	In vivo (Mice): Promotes lumbar spine bone formation, MMP13, ADAMTS4, ADAMTS5↓	[24]
Deer antler	Water	In vivo (Rats): Inhibition of SCW-induced leukocytosis, decrease blood cell adhesion to the endothelium	[67]
Deer antler		Primary chondrocytes: Promoting Cyclin A expression via TK signaling pathway	[81]
Deer Antler	Cold water	Primary chondrocytes: Regulation of multiple growth factors, morphogens and transcription factors	[26]
Deer Antler	Cold water	Primary chondrocytes: Upregulated 192 differentially expressed genes. Promotes chondrocyte proliferation and inhibits apoptosis and differentiation	[27]
Deer Antler	Cold water	In vivo (Rats): Upregulation of DEGs involved in cartilage growth and regeneration, downregulation of DEGs involved in inflammation	[74]

**Table 6:** Effects of DAPs on neurological diseases.

Origin	Extracting Media	Antioxidant Activity	References
Deer Antler	Cold water	SH-SY5Y human neuroblastoma cells: Cell damage was inhibited. Bcl2↑ Bax, Caspase-3↓	[90]
		In vivo (Mice): The number of hippocampal neurons in the brain was restored and neuronal damage in the brain was reduced. CRH, ACTH, CORT, GR, MR↓	
Deer Antler		Nerve cells: Rescue cell damage and apoptosis and promote cell proliferation. Bax, caspase-3↓ Bcl2, p-p38, p-JNK↑	[92]
Deer Antler	Ice-cold acetic acid solution (pH 3.5)	SH-SY5Y human neuroblastoma cells: Alleviates mitochondrial membrane potential collapse, endoplasmic reticulum stress, and elevation of ROS. Inhibits Ca2+-calpain-caspase-12 pathway activation.	[17]
Deer Antler	Hot water	In vivo (Mice): Inhibits morphine-induced analgesic tolerance, somatic dependence, and postsynaptic DA receptor hypersensitivity.	[34]
Deer Antler	Hot water	In vivo (Mice): Repair of presynaptic DA receptor dysfunction and inhibition of postsynaptic DA receptor hypersensitivity.	[33]
Deer Antler	Ice-cold acetic acid solution (pH = 4)	In vivo (Rats): Inhibition of tyrosine hydroxylase positive neuronal death. GAP-43, NF-H↑; Glu, GABA↓	[47]
Traditionally-dried two-branched deer antler	(1) Pepsin solution (pH = 2) (2) Trypsin solution (pH = 7.8–8.5)	In vivo (C. elegans): Increases C. elegans’ longevity and motility and reduces β-amyloid deposition. ROS↓; SOD, skn-1, hsf-1, daf-16, sod-3↑	[43]
Deer Antler		In vivo (Rats): Relieves symptoms of hypoxic-ischemic encephalopathy. HO-1↓ Gpx, Gst, GDNF, NGF, NGFR, SDF1, CXCR4↑	[98]
Deer Antler		In vivo (Rats): Inhibits nerve damage, oxidative stress and inflammation. IL-1β, IL-6, TNFα↓, Nrf-2, HO-1, p-IKKα, p-IKKβ, p-NF-κBp65, p-IκBα↑	[99]
Deer Antler	(1) Cold water (2) Formic acid solution (0.2%)	In vivo (Mice): p-AMPK, Sirt1↑ IL-1β, IL-18, GSDMD-N, NF-κB, NLRP3, ASC, Caspase-1↓	[54]

## Data Availability

Not applicable.
